# Hepatic epithelioid hemangioendothelioma: A diagnostic pitfall in aspiration cytology

**DOI:** 10.4103/1742-6413.58951

**Published:** 2010-01-15

**Authors:** Ruchika Gupta, Sandeep R Mathur, S Datta Gupta, Prashant Durgapal, Venkateswaran K Iyer, Chandan Jyoti Das, Shalimar, Subrat K Acharya

**Affiliations:** Department of Pathology, All India Institute of Medical Sciences, New Delhi, India; 1 Department of Radiodiagnosis, All India Institute of Medical Sciences, New Delhi, India; 2 Department of Gastroenterology and Human Nutrition, All India Institute of Medical Sciences, New Delhi, India

**Keywords:** Aspiration cytology, epithelioid hemangioendothelioma (EH), histopathology, immunohistochemistry, liver

## Abstract

Hepatic epithelioid hemangioendothelioma (EH) is a rare vascular neoplasm. An accurate radiologic diagnosis is usually difficult due to the presence of multiple nodules, simulating metastatic carcinoma. Though histologic features of this tumor are well described, cytologic reports of hepatic EH are very few in the available literature. We describe a case of a young healthy adult male who was found to have multiple hepatic masses on radiologic investigations. A guided fine needle aspiration demonstrated a poorly differentiated neoplasm. The diagnosis was made on core biopsy assisted by immunohistochemistry, which showed characteristic features of EH. He is doing well 14 months after diagnosis, without surgical excision or chemotherapy. An accurate diagnosis of hepatic EH on aspiration cytology requires an adequate specimen and awareness of its cytologic features, including discohesive atypical cells with intracytoplasmic lumina and intranuclear inclusions. Since this tumor is usually unresectable but has a favorable prognosis as compared to hepatocellular carcinoma, a correct diagnosis is essential for appropriate management and prognostication.

## INTRODUCTION

Sarcomas arising primarily in the liver are quite unusual; the most common being angiosarcoma. A rare vascular tumor described in the liver is epithelioid hemangioendothelioma (EH).[1,2] It is a slow-growing vascular neoplasm of intermediate malignant potential and affects young adults. EH is characterized by multifocality of its lesions, which precludes partial hepatectomy. In majority of the cases reported in the literature, the diagnosis was made only on histologic examination of a biopsy or surgical resection specimens.[1–3] Reports of aspiration cytology of this unusual hepatic neoplasm are very few in the available English literature.[4–7] The cytologic features described in these reports are variable, compounding the diagnostic difficulty further.

We describe the cytologic features of a hepatic EH in a young adult male. This rare tumor is briefly discussed along with its distinctive histologic features and various differential diagnoses.

## CASE REPORT

A 23-year-old male presented with a 10-month history of persistent upper abdominal pain associated with loss of appetite. He gave a past history of jaundice 20 months back, which lasted for four weeks with complete resolution thereafter. He was a nonalcoholic, nonsmoker with no comorbid medical/ surgical ailment.

Physical examination showed hepatomegaly with liver palpable 4 cm below right costal margin. The surface of the liver was smoothly lobulated, firm with rounded edges. Systemic examination was unremarkable.

Routine haematological and biochemical investigations, including serum bilirubin, transaminases and proteins were within reference ranges. Serum alkaline phosphatase was 294IU/L. Serological tests for hepatitis B and C was negative. Serum carcinoembryonic antigen, CA 19-9 and alpha-fetoprotein were also normal.

**Figure 1:**
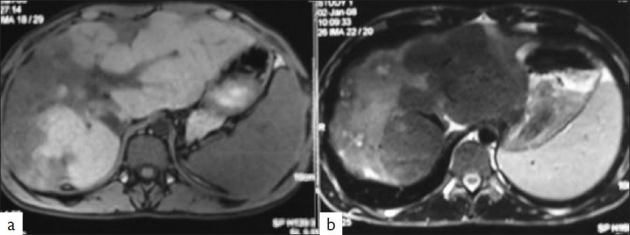
MRI images (T1-weighted) showing a diffuse poorly defined hypointense mass in right and left lobes of liver with subcapsular retraction (a). The mass is hyperintense on T2-weighted image (b)

Contrast-enhanced computed tomography (CECT) scan showed hepatomegaly, more in the left lobe than the right lobe with heterogeneous attenuation and variegated enhancement of right lobe. Magnetic resonance imaging (MRI) confirmed the findings of CECT with ill-defined space-occupying lesions involving both lobes of liver. The mass lesions were hypointense on T1-weighted images and hyperintense on T2-weighted images [Figure 1].

An ultrasound-guided fine needle aspiration (FNA) was performed from one of the hepatic mass lesions. The smears were alcohol-fixed as well as air-dried and stained by Papanicoulaou and May-Grünwald-Giemsa methods, respectively. Due to the limited cellularity on an on-site assessment, an ultrasound-guided trucut biopsy was also performed, simultaneously.

### Cytologic findings

**Figure 2:**
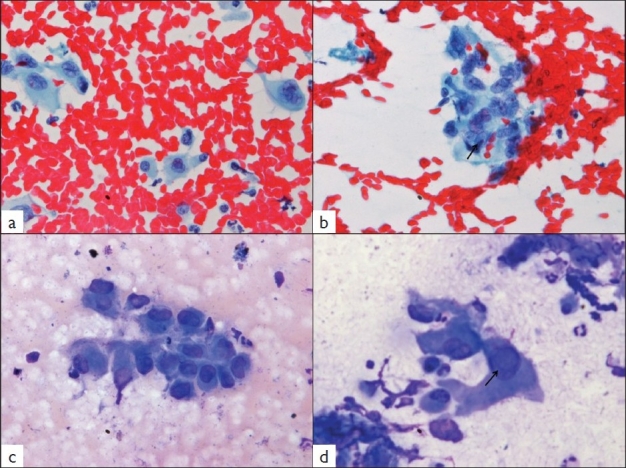
Photomicrographs from aspiration smears from hepatic mass demonstrating singly lying polygonal cells (a, Papanicolaou × 100). A cellular fragment with one cell showing an intranuclear inclusion (arrow) is seen (b, Papanicolaou × 100). The polygonal cells display moderate nuclear pleomorphism (c, May-Grünwald-Giemsa stain × 200). Higher-power view shows an intranuclear inclusion marked by arrow (d, May-Grünwald-Giemsa stain ×400)

The aspiration smears were paucicellular and showed single cells and occasional small tissue fragments [Figure 2a–c]. The cellular component consisted of small bland-appearing polygonal (epithelioid) cells with scant cytoplasm along with few spindle cells. In addition, scattered larger malignant-appearing pleomorphic cells with moderate amount of cytoplasm and hyperchromatic nucleus were noted [Figure 2a]. Occasional cells showed conspicuous nucleoli. Sharply-defined intranuclear pseudoinclusions were noted in many cells [Figure 2b, d]. However, no intracytoplasmic inclusions were seen in the aspiration smears. A few benign bile ductules were also noted. Considering the cytological features, a diagnosis of a poorly differentiated carcinoma was rendered and histological correlation advised.

### Histologic findings

**Figure 3:**
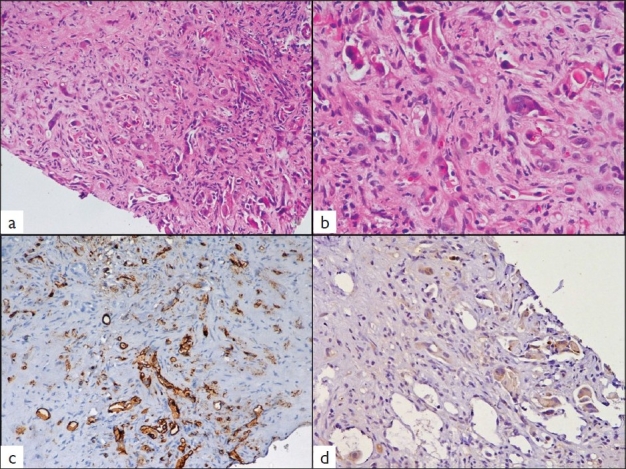
Histological photomicrographs demonstrating singly-lying and cords of cells embedded in a desmoplastic stroma (a, H and E ×100). The tumor cells show intracytoplasmic lumina containing red blood cells (b, H and E ×200). Immunostaining reveals cytoplasmic positivity for CD31 in the tumor cells (c, ×100) and focal positivity for pancytokeratin (d, ×200).

Biopsy section showed a small fragment of liver parenchyma along with fibrous tissue infiltrated by scattered tumor cells [Figure 3a]. The tumor cells were polygonal (epithelioid) with abundant cytoplasm, vesicular nucleus with prominent nucleolus in some cells. Prominent intracytoplasmic vacuoles were seen in many tumor cells [Figure 3b]. An occasional tumor cell showed intracytoplasmic lumina with red blood cells.

Immunohistochemistry revealed the tumor cells to be strongly and diffusely positive for CD31 [Figure 3c] and CD34 with focal reactivity for pancytokeratin [Figure 3d]. The tumor cells were negative for carcinoembryonic antigen, HMB-45 and S-100 protein. Hence, a final histologic diagnosis of hepatic EH was made.

The patient was offered liver transplantation, but he could not afford it. At the last follow-up (14 months after diagnosis), he is well appearing except for mild abdominal pain.

## DISCUSSION

EH is a distinctive vascular tumor occurring mostly in soft tissues of extremities and lung.[3] Involvement of liver may be seen as a metastasis or rarely as a primary tumor.[2] EH has been associated with the use of oral contraceptives, vinyl chloride, Thorotrast and in presence of primary biliary cirrhosis.[4] Hepatic EH occurs in adult life with a mean age at presentation of 41.7 years (12-86 years). Majority of the patients present with non-specific clinical features.[3]

The radiologic findings of hepatic EH usually pose a diagnostic dilemma for the radiologists. Ultrasonography reveals multiple discrete hypoechoic nodules or a diffusely heterogeneous echotexture of the liver. Computed tomography (CT) scan shows multiple discrete nodules with target appearance or a confluent hypodense mass in the liver. Magnetic resonance imaging (MRI) also shows low signal intensity lesions on T1W images and heterogeneous high signal intensity on T2W sequences. Peripheral enhancement and a thin non-enhancing rim are seen on gadolinium-enhanced MRI sequences.[8] These features suggest a diagnosis of metastatic carcinoma and hence, radiologic diagnosis may not be possible, as also seen in the present case. However, some authors have called attention to the discordance between radiologic features and good general condition and long history of symptoms.[3]

In the absence of classic radiologic features, pathologic examination remains the mainstay of diagnosis of this rare tumor. Currently there is an increasing use of guided fine needle aspiration cytology (FNAC) in diagnosis of accessible visceral lesions, especially hepatic masses. As a result, rare tumors are being subjected to FNAC, and hence, cytopathologists should be aware of the cytologic picture of unusual tumors, like EH. The present case, on review and correlation with histomorphology, depicts most of the cytologic features of hepatic EH. As described in the earlier reports,[4–7] we observed low cellularity of the smears comprising of singly dispersed cells and small tissue fragments. The tumor cells had variable cytomorphology, varying from epithelioid or spindled character to scattered larger bizarre cells with hyperchromatic nuclei. Occasional cell showed intranuclear inclusion, similar to those described in EH of soft tissue.[9] On the other hand, intracytoplasmic vacuoles or lumina, seen very well in histology, are rarely seen in cytology. In retrospect, preparation of cell blocks with the small amount of aspirate would help in making an accurate diagnosis on cytology assisted by immunocytochemistry.

The various cytologic differential diagnoses in a smear from hepatic EH would include hepatocellular carcinoma, cholangiocarcinoma, metastatic carcinoma, malignant melanoma and angiosarcoma. Of these, hepatocellular carcinoma can be excluded by the absence of characteristic vascular network, trabecular pattern of tumor cells and the presence of normal hepatocytes in the background. Cholangiocarcinoma is ruled out by the finding of normal bile ducts in the background, as seen in the present case. The low cellularity, absence of prominent eosinophilic nucleoli and pigment in the tumor cells helps in excluding melanoma. Smears from metastatic carcinoma are usually hypercellular composed of pleomorphic cells with prominent nucleoli and cytoplasmic vacuoles containing mucin in some cases. In addition, the good general condition in the presence of extensive hepatic involvement militates against the diagnosis of metastatic carcinoma.[3] Cytologic distinction from other tumors can be assisted by preparation of cell block and immunocytochemistry for endothelial markers in a panel of antibodies.

The histopathologic appearance of hepatic EH comprises of round or spindle cells embedded in a fibrous stroma in the central part of the tumor. The tumor cells have a propensity for invasion of terminal hepatic venules and portal vein branches.[1] The tumor is composed of pleomorphic cells with rare multinucleate giant cells. The vascular nature of the tumor may be apparent only by an occasional cell showing intracytoplasmic lumina formation containing erythrocyte or leukocyte. The endothelial nature of tumor cells may be confirmed by the immunohistochemical detection of CD31, CD34, factor VIII-related antigen or Ulex europeus 1 lectin.[4] Ultrastructural demonstration of Weibel-Palade bodies within the tumor cells also confirms the endothelial nature.[1]

The biologic behaviour of this tumor is that of a slow-growing neoplasm with long survival. Hepatic EH has a low propensity to metastasize. Since the tumor is relatively avascular, there is little risk of life-threatening rupture. However, in a few patients, a rapidly progressive course with fatal outcome has been reported.[10,11]

Orthotopic liver transplantation appears to be the only hope for patients with hepatic EH, since surgical resection is impossible due to the multifocality of the tumor.[1,3] Many of the reported patients have undergone successful transplantation with survival to seven years.[2] Chemotherapy is usually ineffective in hepatic EH.[2] In the present case, the patient could not afford transplantation, hence is being followed up regularly.

In conclusion, EH is an extremely rare primary hepatic tumor. With the use of aspiration cytology in diagnosis of hepatic masses, the cytopathologists should be aware of the cytologic features of this unusual neoplasm. The use of ancillary techniques like cell-block preparation with immunohistochemistry is imperative in making an accurate diagnosis on cytology.

## COMPETING INTEREST STATEMENT BY ALL AUTHORS

No competing interest to declare by any of the authors.

## AUTHORSHIP STATEMENT BY ALL AUTHORS

All authors of this article declare that we qualify for authorship as defined by ICMJE http://www.icmje.org/#author.

Each author has participated sufficiently in the work and take public responsibility for appropriate portions of the content of this article.

Each author acknowledges that this final version was read and approved.

## ETHICS STATEMENT BY ALL AUTHORS

This study was conducted with approval from Institutional Review Board (IRB) (or its equivalent) of all the institutions associated with this study. Authors take responsibility to maintain relevant documentation in this respect.
